# Readout IC Architectures and Strategies for Uncooled Micro-Bolometers Infrared Focal Plane Arrays: A Review

**DOI:** 10.3390/s23052727

**Published:** 2023-03-02

**Authors:** Samuele Fusetto, Antonio Aprile, Piero Malcovati, Edoardo Bonizzoni

**Affiliations:** Department of Electrical, Computer and Biomedical Engineering, University of Pavia, 2100 Pavia, Italy

**Keywords:** micro-bolometers, bolometers, IRFPA, readout interfaces, thermal imaging

## Abstract

InfraRed Focal Plane Arrays (IRFPAs) are crucial components in a wide range of applications, including night vision, thermal imaging and gas sensing. Among the various types of IRFPAs, micro-bolometer-based ones have gained significant attention due to their high sensitivity, low noise and low cost. However, their performance is heavily dependent on the readout interface, which converts the analog electrical signals provided by the micro-bolometers into digital signals for further processing and analysis. This paper briefly introduces these kinds of devices and their function, reporting and discussing a list of key parameters used to evaluate their performance; after that, the focus is shifted to the readout interface architecture with particular attention to the different strategies adopted, across the last two decades, in the design and development of the main blocks included in the readout chain.

## 1. Introduction

Uncooled micro-bolometer InfraRed Focal Plane Arrays (IRFPAs) are a type of infrared detector that is commonly used in thermal imaging applications. It consists of a two-dimensional array of micro-bolometers, which are tiny thermally sensitive resistors that are used to detect the infrared radiation emitted by objects in the detector’s field of view; these sensing elements are placed on a silicon substrate and are typically fabricated using a Micro-Electro-Mechanical System (MEMS) technology. This type of detectors offer a relatively high spatial resolution and sensitivity; this allows the arrays that compose them to accurately detect and distinguish between objects with small temperature differences. Another key advantage is that they do not require any external cooling; this makes the thermal imaging system more portable, easier to use and ideal to be employed in a large variety of fields. For example, in military and security applications, micro-bolometer IRFPAs can be used to detect the presence of humans or vehicles in low light or obscured visibility conditions, such as fog, smoke or darkness [1,2,3,4,5,6,7,8,9,10,11,12,13]. In the industrial and building inspections framework, they can be exploited to identify and diagnose problems in electrical systems, mechanical equipment and building insulation, whereas, in leak detection, they can be used to identify and locate leaks in pipes, roofs and other structures [1,2,3,5,12,14,15,16,17]. In the medical field, instead, they can be employed to detect and monitor temperature changes in the human body, such as in fever screening or as a diagnostic tool for various medical conditions [1,3,4,6,8,12,14,15,16,17,18,19]. Moreover, in agriculture, they can be used to detect crop stress, identify pests and monitor irrigation efficiency [20]; in addition, in the environmental framework, they can be exploited to monitor the health and status of wildlife, detect changes in land and water temperatures and monitor the performance of renewable energy systems, such as solar panels [1,17,18]. Furthermore, in the automotive industry, they can be used to detect problems with a car’s engine, transmission or brakes and for pedestrian detection and night vision [5,10,12,21,22]. Finally, in research and development, they can be adopted to study and understand the heat transfer and the thermal behavior of materials and systems [12,13,15].

A key block of thermal imaging systems based on micro-bolometers IRFPAs is the ReadOut Integrated Circuit (ROIC): it provides the bias for each micro-bolometer and converts the resulting information into a signal that can be fed to a video amplifier or to an Analog-to-Digital Converter (ADC). The ROIC also houses the circuitry required to compensate for the self-heating effect given by the biasing and for pixel non-idealities due to fabrication process variations. This paper briefly introduces micro-bolometers and their function, reporting and discussing a list of key parameters used to evaluate their performance; after that, a review of the commonly used readout interface architectures is presented with a focus on the strategies adopted, across the last two decades, in the design of the main blocks included in the readout chain.

## 2. Micro-Bolometers: Device Structure and Operation Principle

The considered thermal-imaging sensors consist of a matrix of pixels, each of which is composed of a micro-bolometer, interconnections and part of the readout structure such as multiplexing switches. The micro-bolometer absorbs InfraRed (IR) radiation and this changes its electrical resistance; this variation is then detected by an interface circuit to create a thermal image of the scene in front of the camera. The pixel matrix is typically fabricated using a MEMS process on a Complementary Metal-Oxide-Semiconductor (CMOS) substrate that houses the interconnections and the required Integrated Circuits (ICs); over the years, various techniques have been developed to obtain micro-bolometers with an increasing Temperature Coefficient of Resistance (TCR) (this parameter will be addressed in detail in Section 3), with a reduced thermal conductivity and with the goal of a noise minimization.

**Figure 1:**
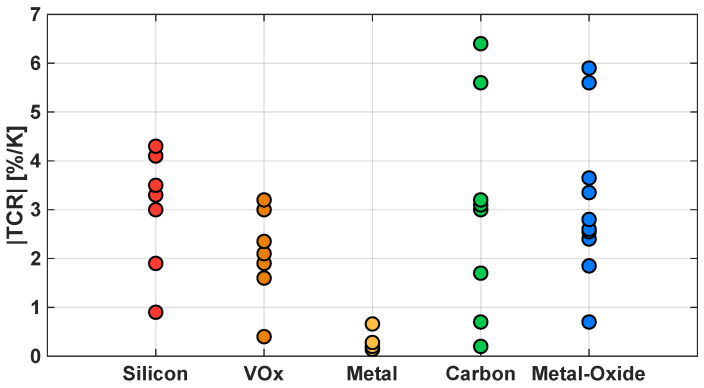
TCR values of commonly employed material types for the micro-bolometer’s sensing membrane.

The pixel structure can be divided into three parts: the bolometer membrane, the support structures and the cavity. The bolometer membrane is the sensing element, and its material determines its capabilities. Amorphous Silicon (a-Si) and Vanadium Oxide (VO_x_) are the most commonly used materials due to their high TCR and compatibility with standard CMOS and MEMS integration processes. However, research has been conducted in recent years to find new materials, such as graphene and Carbon NanoTube (CNT) composites, or different types of metal-oxides, such as Titanium Oxide (TiO_x_), that can improve the micro-bolometer’s performance. Metal-based membranes, which typically exhibit a lower TCR, instead offer the advantage of an easier fabrication process, which results in a lower manufacturing cost. Figure 1 summarizes the TCR values for these different categories of materials according to [12].

**Figure 2:**
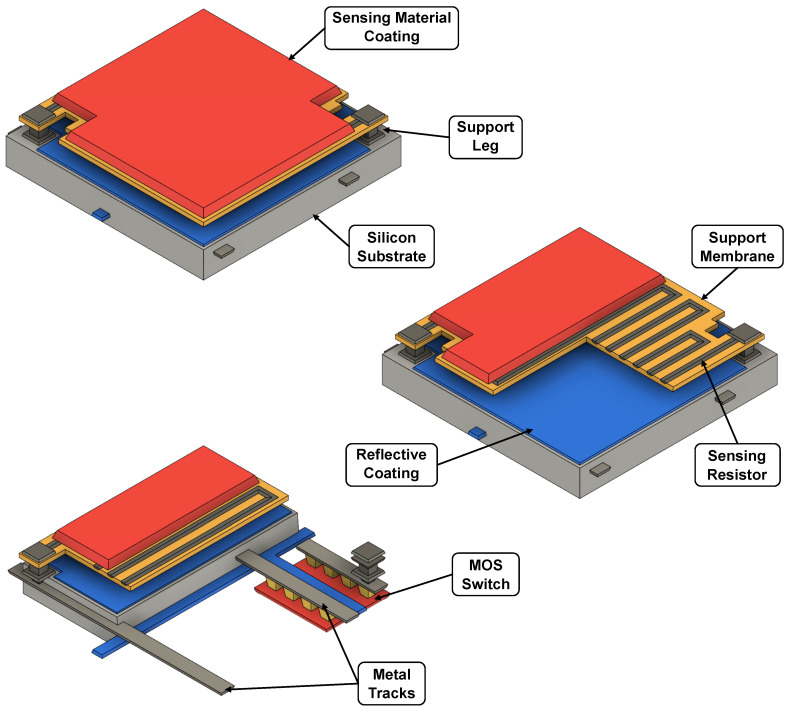
Three-dimensional layout of a micro-bolometer with a breakdown of its main parts.

**Figure 3:**
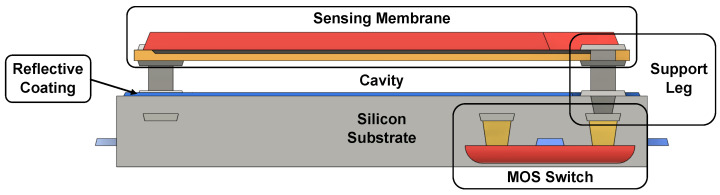
Micro-bolometer cross-section.

A 3D layout of the described micro-bolometer structure is shown in Figure 2, while Figure 3 reports its cross-section.

In order to reduce heat flow, the sensing membrane needs to be suspended; this is achieved through the use of various types of supporting structures known as legs. In earlier micro-bolometers, the sensing layer was directly deposited on the substrate, and a cavity was then etched beneath it: this meant that all the necessary interconnections and devices to bias and read the pixel had to be integrated beside the micro-bolometer, leading to a poor filling factor. With the advancement of MEMS fabrication processes, a two-level structure, in which the micro-bolometer is separated from the substrate, has become possible, as depicted in Figure 2 and Figure 3; with this arrangement, all the required interconnection circuitry can be integrated into the empty space under the pixel, and heat flow is only possible through the supporting legs, which also serve as an electrical connection between the sensing element and the rest of the system. Another consequence of the MEMS technological development is the possibility to create compact, robust and low thermal conductance supporting structures; in addition, it is now possible to completely separate the IR absorber from the sensing membrane by creating an umbrella structure (not shown) above it, which maximizes the filling factor [23]. The cavity between the sensing membrane and the substrate is used to reduce heat flow; for this reason, the entire detector is enclosed in a rigid metal package under vacuum. The cavity can also be used to enhance the absorbance of the micro-bolometer by controlling its height; if the distance between the sensing membrane and the substrate is chosen to be equal to $\lambda_{IR}\;/\;4$, where $\lambda_{IR}$ is the infrared wavelength of interest, the void acts as a Fabry–Perot optical cavity and this increases the performance of the micro-bolometer. To maximize this effect, the substrate under the sensing plate is coated with a reflective layer, typically aluminum, and the top of the membrane is covered with an anti-reflective material.

Defining the sensing membrane resistance as

(1) $$R=R_{0}\left(1+\alpha \Delta T\left(t\right)\right),$$

where $R_{0}$ is its room temperature value, α corresponds to the previously introduced TCR parameter and ΔT is the temperature difference between the sensing membrane absorbing the IR radiation and the rest of the device, the micro-bolometer’s behavior can be described by the heat balance equation [24,25]

(2) $$C\frac{d\Delta T(t)}{dt}+G\Delta T\left(t\right)=\underset{applied\;heat\;flow}{\underbrace{\eta \beta A_{D}P_{rad}+I_{b}^{2}\left(t\right)R}}.$$

The left-hand side represents the heat present in the micro-bolometer, which is influenced by heat conduction (*G*) and heat capacity (*C*). The right-hand side, instead, represents the heat applied to the micro-bolometer, which can come from external radiation ($P_{rad}$) or from the bias current ($I_{b}$) of the device. The η and β constants are the absorption coefficient and the filling factor of the micro-bolometer array, respectively, while $A_{D}$ is the pixel area. Equation (2) allows the analysis of the impact of the incident radiation, of the substrate thermal leak and of the biasing current on the micro-bolometer temperature; even if all resistive micro-bolometers need to be biased, it is useful to solve it considering only the effect of the power ($P_{rad}$) of a modulated (at a ω angular frequency) incident radiation while imposing $I_{b}=0$:

(3) $$\Delta T\left(t\right)=\frac{\eta \beta A_{D}P_{0}}{G{\left(1+\omega^{2}\tau^{2}\right)}^{1\;/\;2}}.$$

(4) $$\tau =\frac{C}{G},$$

(5) $$P_{rad}=P_{0}exp\left(j\omega t\right),$$

where τ is called the thermal time constant (it is measured in seconds) while $P_{0}$ is the power amplitude of the incident radiation. Equation (3) shows that the increase in temperature due to the incident radiation is inversely proportional to the thermal conductance of the micro-bolometer (*G*) that takes the heat flow through the supporting legs and the power radiated back by the micro-bolometer into account. This remarks the importance of good thermal isolation of each pixel; typically, the thermal effects of the biasing current and substrate are much larger than the infrared radiation, so they need to be correctly compensated or suppressed.

## 3. Parameters of Interest

In this section, a list of the most useful parameters related to the micro-bolometers framework will be discussed.

### 3.1. Temperature Coefficient of Resistance (TCR)

The TCR of a micro-bolometer relatively indicates how the resistance (*R*) of the employed sensing material changes with temperature and is measured in is 1/K. It is given by:

(6) $$TCR=\alpha =\frac{dR}{RdT}$$

where α is the coefficient introduced in (2).

In the case of semiconductor micro-bolometers, the TCR is typically a negative value, which means that the resistance decreases as the temperature increases; moreover, it is strictly dependent on the material used to coat the sensing membrane of the micro-bolometer, as previously reported in Figure 1.

### 3.2. Responsivity (R)

The responsivity (R) of a micro-bolometer is a measure of the device’s efficiency at converting incident radiation into an electrical signal. It is typically expressed in units of volts per watt (V/W), and it is computed as the ratio of the output voltage signal ($V_{sig}$) and the incident radiation power ($P_{0}$) falling on the pixel:

(7) $$R=\frac{V_{sig}}{P_{0}}.$$

The voltage generated by the flow of the bias current ($I_{b}$) through the micro-bolometer is straightforwardly given by:

(8) $$V_{out}=\underset{V_{b}}{\underbrace{I_{b}\cdot R_{0}}}+\underset{V_{sig}}{\underbrace{I_{b}\cdot \alpha \Delta TR_{0}}}.$$

Combining the $V_{sig}$ expression of (8) with (3), a basic formula for R can be derived:

(9) $$R=\frac{I_{b}\cdot \alpha \Delta TR_{0}}{P_{0}}=\frac{I_{b}\cdot \alpha R_{0}\cdot \eta \beta A_{D}}{G_{0}{\left(1+\omega^{2}\tau^{2}\right)}^{1\;/\;2}}.$$

Accurately computing the responsivity parameter in resistive micro-bolometers, nevertheless, is definitely challenging due to the complexity introduced by the biasing current in the heat balance equation; on top of that, in most cases, in order to reduce self-heating effects and to take advantage of multiplexing strategies (discussed in Section 4) the applied bias is not constant but pulsed.

### 3.3. Noise

In the micro-bolometers framework, four sources of noise can be identified: Johnson noise (thermal), 1/f noise (flicker), temperature fluctuation noise and background noise [25].

1. The Johnson noise ($V_{N,J}$) arises due to the random thermal motion of charge carriers in the micro-bolometer’s sensing material, and it increases as the temperature of the device rises; its Power Spectral Density (PSD) can be expressed as
  (10) $$\bar{V_{N,J}^{2}}=4kTR,$$
  where *k* is Boltzmann’s constant, *T* is the temperature of the micro-bolometer and *R* is its resistance as introduced in (2).
2. The 1/f noise ($V_{N,1\;/\;f}$) is caused by various factors, such as impurities in the materials used to fabricate the micro-bolometer or by defects in the device itself, and it is characterized by a PSD that decreases with increasing frequency according to
  (11) $$\bar{V_{N,1\;/\;f}^{2}}=\frac{\alpha_{H}\cdot V_{out}^{2}}{f},$$
  where $V_{out}$ was defined in (8), and $\alpha_{H}$ is a dimensionless constant called the Hooge parameter [26].
3. The temperature fluctuation noise ($V_{N,TF}$), instead, is caused by small changes in the temperature of the micro-bolometer detector that occurs due to changes in the environment or by power fluctuations in the device itself. Its PSD can be expressed as
  (12) $$\bar{V_{N,TF}^{2}}=\frac{4kTGR^{2}}{\eta },$$
  where all the variables have the same meaning as introduced before.
4. The background noise ($V_{N,BG}$) refers to any unwanted signal falling on the detector and can be caused by various factors such as ElectroMagnetic Interference (EMI) from other devices or by the detector’s own ROIC (it is also called radiation noise). Its PSD can be expressed as
  (13) $$\bar{V_{N,BG}^{2}}=8A_{D}\eta \sigma k\left(T_{D}^{5}+T_{B}^{5}\right)R^{2},$$
  where $T_{D}$ is the pixel temperature, $T_{B}$ is the background temperature, and σ is Stefan’s constant.

Since these types of noise are uncorrelated, it is possible to define a total noise voltage ($V_{N,tot}$) as the square root of the quadratic sum of all these sources according to

(14) $$V_{N,tot}=\sqrt{\bar{V_{N,J}^{2}}+\bar{V_{N,1\;/\;f}^{2}}+\bar{V_{N,TF}^{2}}+\bar{V_{N,BG}^{2}}}.$$

In conclusion, considering that, usually, an integrator is used when a pulsed bias is applied to the micro-bolometer, the noise bandwidth is limited by the inverse of the integration time ($t_{int}$):

(15) $$B=\frac{1}{2t_{int}}.$$

### 3.4. Noise at Equivalent Temperature Difference (NETD)

**Figure 4:**
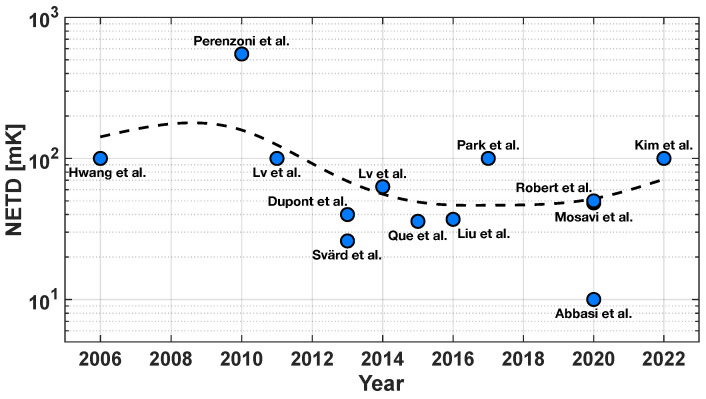
NETD trend of the main micro-bolometer works of the last 17 years [1,2,3,5,14,16,17,21,22,27,28,29,30].

The NETD parameter is a measure of the sensitivity of a micro-bolometer. It corresponds to the smallest temperature difference between two objects in the same scene that the sensor can detect: the lower the NETD value, the higher the sensitivity of the micro-bolometer. It is defined as

(16) $$NETD=\frac{4F^{2}V_{N,tot}}{\tau_{o}A_{D}R{\left(\Delta P/\Delta T\right)}_{\lambda_{1}-\lambda_{2}}},$$

where *F* and $\tau_{o}$ are parameters that take the optics of the system into account; ${\left(\Delta P/\Delta T\right)}_{\lambda_{1}-\lambda_{2}}$ is the change with respect to the temperature of the power per unit area emitted by a blackbody within the spectral band form $\lambda_{1}$ to $\lambda_{2}$, and it is measured in Kelvin [25]. Considering the great relevance of this parameter in this framework, Figure 4 reports the NETD trend of the last 17 years, taking the main micro-bolometer works published in this timeframe into account [1,2,3,5,14,16,17,21,22,27,28,29,30]; the dotted curve has been obtained by a log-scale adapted smoothing spline method [31]. It can be noticed that the trend line exhibits a negative slope across the 2010–2016 period; however, this behavior extends no further since, in typical applications, a NETD value in the order of 100 mK is considered sufficient.

### 3.5. Noise at Equivalent Power (NEP)

NEP is a parameter used to estimate the power introduced in the system by the various noise sources, and it is defined as the amount of input power necessary to have a signal-to-noise ratio of one. It is typically computed according to

(17) $$NEP=\frac{V_{N,tot}}{R}$$

and it is measured in $W\;/\;\sqrt{Hz}$.

### 3.6. Detectivity ($D^{*}$)

The detectivity is a metric mostly used to compare micro-bolometers with different pixel areas ($A_{D}$) and with different operating bandwidths. It can be expressed as the inverse of the NEP scaled by the square root of the pixel dimension and bandwidth,

(18) $$D^{*}=\frac{\sqrt{\beta A_{D}\cdot B}}{NEP},$$

and it is typically measured in $cm\sqrt{Hz}\;/\;W$.

NETD, NEP and $D^{*}$ all depend on R; this means that the accuracy of the reported expressions is related to the assumption made about the heat balance equation in Section 2; completely neglecting the effect given by the bias current, indeed, can adversely affect the noise performance estimation. A complete analysis of this topic is out of the scope of this review; extra information can be found in [11,13,32,33].

## 4. ROIC Architectures

As introduced before, the ROIC is a crucial component of the considered thermal imaging systems. It houses all the necessary blocks to bias the micro-bolometer, extract the resistance variation caused by an incident IR wave (which is the information to be read) and digitize it for post-processing, display and storage.

**Figure 5:**
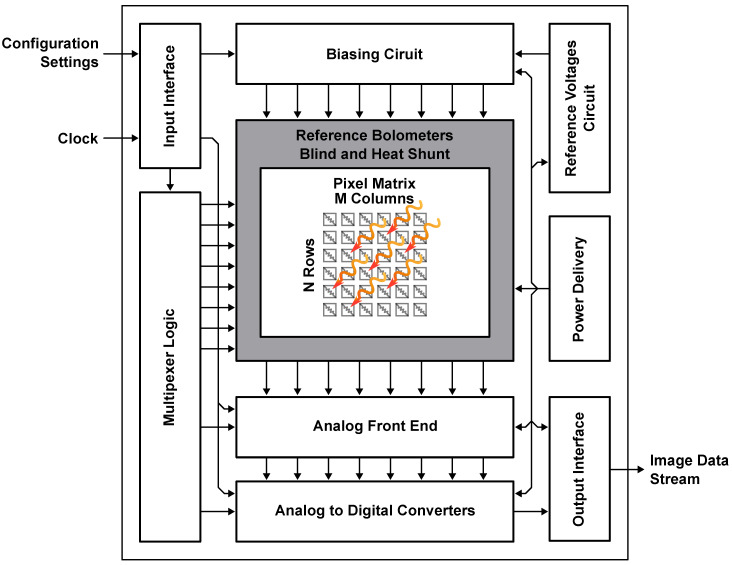
Conceptual block diagram of an IRFPA ROIC.

A conceptual block diagram of an IRFPA ROIC is illustrated in Figure 5. It includes an Input Interface that connects external signals to the system, a Multiplexer Logic that controls the read order of the micro-bolometer-based pixels and a Biasing Circuit that provides the necessary electrical bias to all of the pixels. Additionally, a Reference Voltage Circuit generates a Zero-Temperature-Coefficient (ZTC) voltage used in the other blocks and an Analog Front End (AFE) circuit amplifies and filters the signals generated by the micro-bolometers matrix (NxM). The analog signals are then converted to digital data by means of an ADC, whose output is finally provided to external devices through an Output Interface. Lastly, a Power Delivery unit supplies all the circuits present in the ROIC, whose main blocks (Biasing Circuit, AFE, Multiplexer Logic, ADC) will be discussed in the following subsections.

### 4.1. Biasing Circuit

A micro-bolometer can be seen as a temperature-sensing resistor; its resistance change, caused by an incident IR wave, can be extracted in two ways: with a voltage mode approach, fixing the current flowing in the resistor and reading the voltage at its terminals, or with a current mode approach, fixing the voltage at its terminals and reading the current through it. In both cases, the bias is typically applied for a short time ($t_{int}$) to each micro-bolometer of the detector’s matrix in accordance with the multiplexing operation that is needed to perform the reading of all pixels (discussed in Section 4.3); this allows each micro-bolometer to cool down after being heated by the biasing process.

In both reading modes, blind micro-bolometers [17,30,34], heat shunt micro-bolometers [3,21,28] or a combination of the two are used as a reference in the majority of the compensation strategies implemented to minimize the self-heating and the substrate temperature ($T_{sub}$) variation effects. The blind micro-bolometer has the same structure as the sensing ones, but after its fabrication, it is coated with a reflective film that blocks all the IR radiation; in this way, its resistance change depends only on the other sources of heat. Heat shunt micro-bolometers, instead, are constructed to have a high thermal conductance with the substrate (exactly the opposite with respect to the sensing micro-bolometer); in this way, their temperature is not affected by the bias and can be employed as a reference.

As previously mentioned, the main signal generated by the incident IR radiation, whether it is a voltage or a current, can be affected by undesired effects, such as bias self-heating, substrate temperature or fabrication non-uniformity. All these non-idealities result in a Fixed Pattern Noise (FPN) [35] that degrades the performance of the detector. Usually, the fabrication of non-uniformity given by process variations can be compensated for with the use of shutters and calibration algorithms [2,5,10,29,36,37]; for the remaining undesired effects, instead, a frame-by-frame approach is generally preferred. One of the main strategies to reduce the FPN, which can be applied both for the voltage and the current reading modes, is the Bias EQualization (BEQ) method [38]. The BEQ strategy is based on the control of the biasing of each micro-bolometer of the pixel matrix and typically relies on a three-stage non-uniformity correction algorithm. During the first stage, the substrate temperature non-uniformity effects are corrected for all the detector’s elements; then, traditional offset and gain non-uniformity correction stages are carried out.

**Figure 6:**
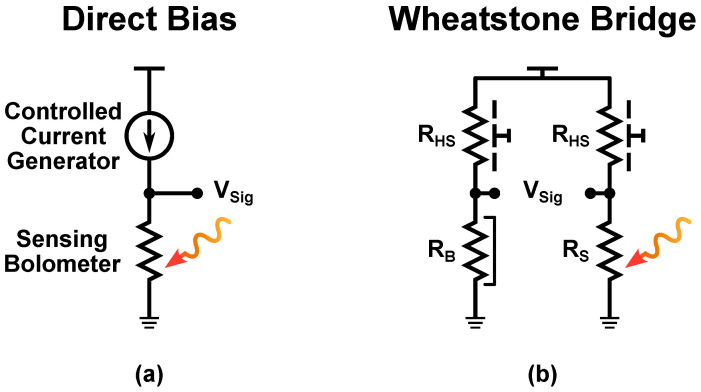
Commonly used voltage mode readout circuits: direct bias approach (**a**) and Wheatstone bridge approach (**b**).

The most simple voltage mode readout structures are based on the use of a current generator to directly bias the micro-bolometer [2,10,14,18,39], as shown in Figure 6a, or on a Wheatstone bridge [40], as shown in Figure 6b. In the first case, the generator used to bias the sensing device does not provide a constant current, which is instead digitally controlled [2,5,10] or generated by analog circuits that make it dependent on $T_{sub}$ [14], in order to compensate for the FPN. When a Wheatstone bridge is used, on the other hand, a heat shunt and a blind micro-bolometer are employed in combination with the sensing one; this allows the compensation for both self-heating and substrate temperature non-uniformity effects.

**Figure 7:**
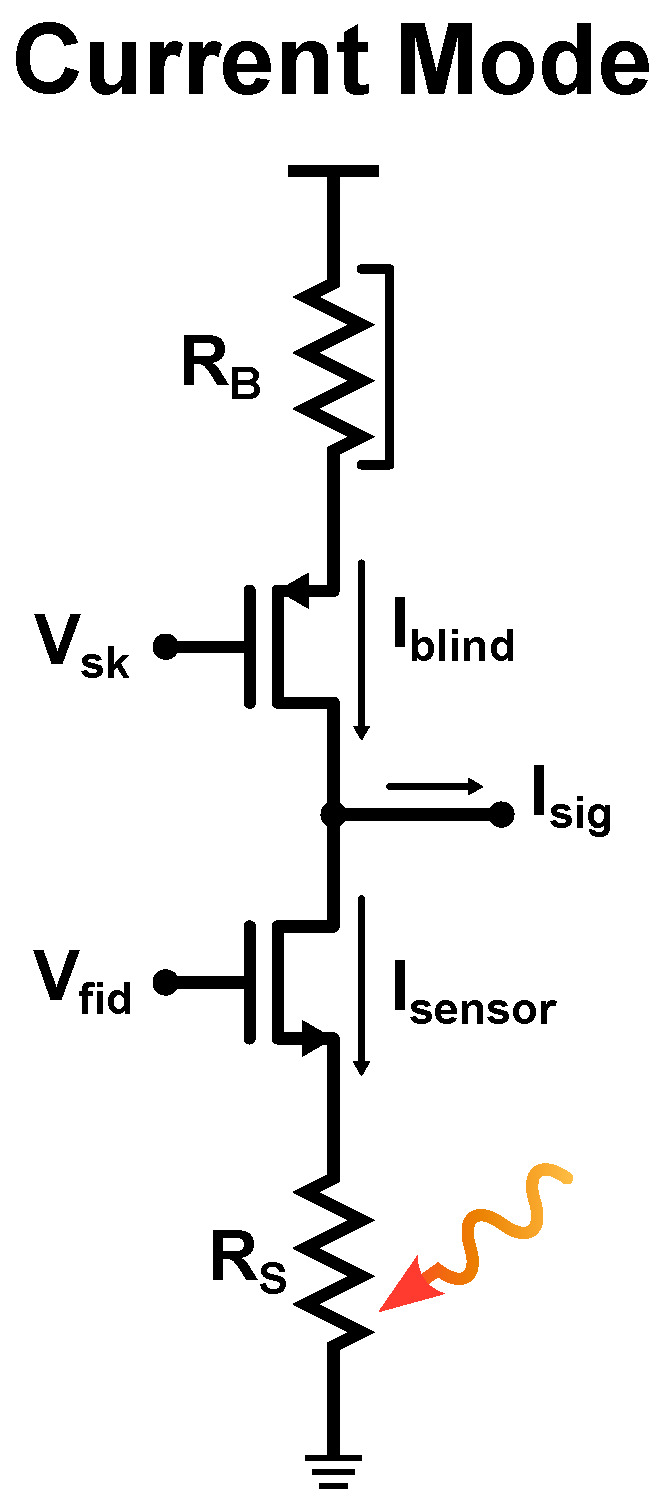
Commonly used current mode readout circuit.

Actually, a current mode readout is adopted in the majority of the ROICs presented in the literature [1,3,4,8,15,17,19,21,27,29,30,34,36,41,42,43,44,45], most of which are based on the circuit shown in Figure 7.

In this scheme, $V_{sk}$ and $V_{fid}$ are used to control the voltage drop across the sensing micro-bolometer $R_{S}$ and across a reference one $R_{B}$, which is usually blinded. The output node is usually clamped at a fixed voltage thanks to the virtual short-circuit of the cascaded Capacitive TransImpedence Amplifier (CTIA), which acts as an integrator. Considering the current flowing through each micro-bolometer, the following equations can be derived:

(19) $$I_{sensor}=I_{DC}+I_{sh}+I_{sub}+I_{rad},$$

(20) $$I_{blind}=\frac{I_{DC}}{1+M}+\frac{I_{sh}}{1+M}+\frac{I_{sub}}{1+M}.$$

$I_{DC}$ is the contribution given by the needed biasing, $I_{sh}$ is due to the self-heating effect, $I_{sub}$ is the component caused by the substrate effect and $I_{rad}$ is the signal generated by the incident radiation to be read. In addition, the *M* parameter represents the matching between the two micro-bolometers; if M=0, the output current is only given by the desired current signal ($I_{rad}$), with all the undesired effects completely cancelled. However, this is not possible due the unavoidable presence of Process Voltage Temperature (PVT) and mismatch variations affecting the considered components and contributing to the FPN; for this reason, $V_{sk}$ and $V_{fid}$ are regulated to compensate for these effects. These control voltages can be generated by means of Digital-to-Analog-Converters (DACs) [6,28,29] or by means of purely analog circuits [1,3,15,17,21,30].

Another commonly used strategy to reduce the FPN is the Correlated Double Sampling (CDS) [6,9,16,41,46] technique; it is used in imaging sensors, such as those present in digital cameras, to reduce noise in the images generated as output. The basic idea is to take two samples of the sensor’s output: one with the sensor’s exposure enabled and one with the exposure disabled; the two samples are then subtracted, effectively cancelling out FPN and other types of noise that are common to both samples. This results in a cleaner and higher-quality image and makes the CDS method widely adopted in Charge-Coupled Devices (CCD) and CMOS image sensors. In addition, Multiple Digital CDS (MD-CDS) is an upgrade of the traditional CDS technique that aims to further reduce the noise of imaging sensors; instead of taking just two samples, MD-CDS takes multiple images and processes them according to [28,47] to achieve further noise reduction.

### 4.2. Analog Front End (AFE)

The AFE has the main function of boosting the signal to be processed by the ADC; generally, it provides a variable gain and integrates the input signal in order to reduce the micro-bolometers’ noise. As introduced in (15), the integration time ($t_{int}$) is a key parameter since it defines the noise bandwidth; this means that a trade-off between the noise and reading time exists. With a lower $t_{int}$, the noise turns out to be higher, but the limit on the number of pixels to be read within the frame time ($t_{frame}$) is less stringent; on the contrary, with a larger $t_{int}$, the noise bandwidth gets reduced, but the degree of needed parallelism turns out to be higher for a given $t_{frame}$, typically resulting in a larger occupied area and higher power consumption. In addition, the AFE, in combination with the previously discussed biasing circuit, is used to implement the necessary functions to carry out the correction strategies needed to reduce the FPN, such as gain [2,34,48] and offset compensation or part of the CDS algorithm [16]. In the case of a voltage mode readout, the AFE is generally made up of a differential amplifier [2,14] or of a capacitive amplifier [5] followed by an integration stage. In most of the current mode readouts, instead, the AFE is composed of a CTIA integrator [1,4,6,8,9,16,17,27,30,34,39,41,42,45,49], sometimes preceded by a pre-amplification stage [17]. The CTIA integrator allows the conversion of the current signal coming from the previous block and provides the fixed voltage needed in the biasing circuit, thanks to the virtual short circuit obtained by means of negative feedback. Independently from the readout mode, some of the AFEs make use of the chopping technique [5,29] in order to reduce flicker noise (1/f noise) and to improve, in general, the total noise performance of the ROIC.

### 4.3. Multiplexing Strategies

**Figure 8:**
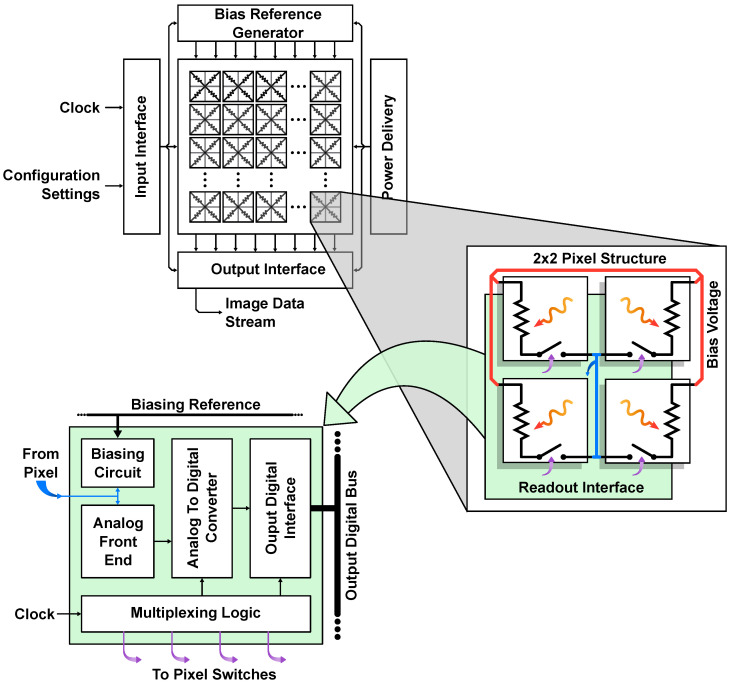
Pixel-wise readout scheme with a magnified view of the pixel interface.

**Figure 9:**
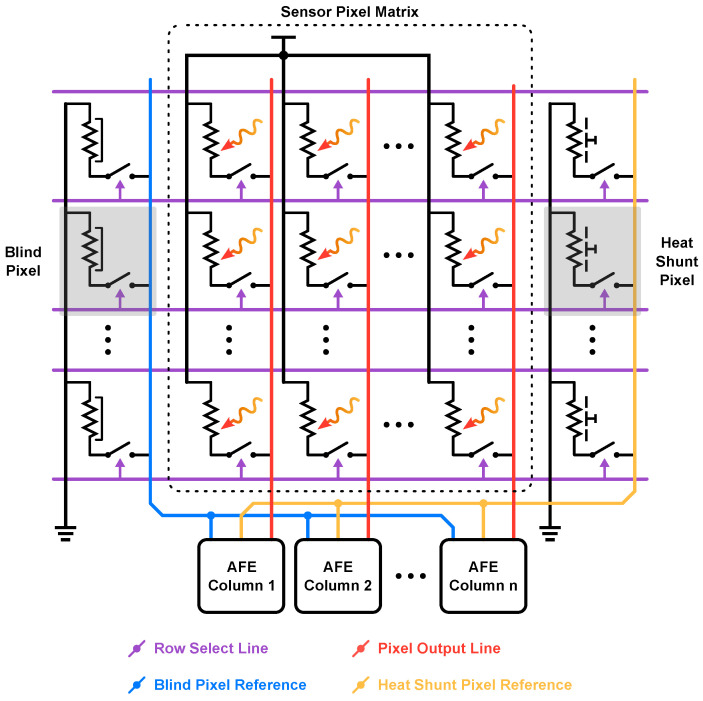
Column-wise readout scheme with a focus on the micro-bolometer pixel matrix interconnections.

All the pixels that compose the IRFPA need to be read within a certain frame time ($t_{frame}$); in order to do this, three different techniques can be identified depending on the adopted multiplexing method:

- Pixel-wise readout: as shown in Figure 8, in correspondence of each pixel (or set of pixels, for instance 2 × 2), a copy of the readout chain is placed. This type of architecture allows the use of the full $t_{frame}$ to read the information from each pixel (or set), which results in a long integration time, reducing the noise bandwidth. However, integrating the whole necessary circuitry within the area of a single pixel, which is typically between 17 and 40 µm^2^, is not always possible. To alleviate this restriction, one single readout chain can be used for more than one pixel (usually four pixels in a 2 × 2 pattern, as shown in Figure 8) to match the area requirement [42,43,44,45].
- Column-wise readout: as shown in Figure 9, each column of pixels shares the AFE and, with the use of some switches, all the pixels in a single row are read at the same time (rolling shutter mode); in this way all the rows are sequentially read to form the frame. With this approach, the bulk of the readout circuitry is heavily parallelized, and the device area and power consumption are reduced. The main disadvantages of this type of technique are derived from the reduced amount of time available for each pixel; as discussed before, this directly translates in a lower $t_{int}$, which increases the noise bandwidth [2,5,14,28].
- Serial readout: according to this readout structure, a single readout chain is exploited. Each pixel is connected to it and is read in a serial fashion. Nevertheless, this type of structure is no longer adopted since the trend in the IR sensors framework is to increase the number of pixels, keeping the frame rate constant; accordingly, the serial approach features a very small time interval dedicated to each pixel, making the requirement on the ROIC extremely stringent.

In conclusion, column-wise multiplexing is the method that balances the advantages and disadvantages given by the two other approaches (pixel-wise and serial) and, therefore, is the most used technique.

### 4.4. Analog-to-Digital Converter (ADC)

The ADC is the block that receives the sampled output of the AFE as input and generates IR-dependent digital words as output. There are many ADC types and architectures to be used in the micro-bolometer framework. One of the simplest ways, for instance, is to use the integrator already present in the system (both with a voltage or a current mode approach) to implement a single-slope A/D conversion [3,14,17,50]. The output ramp of the integrator is compared with a reference voltage; a change in the input signal impacts the slope of the output ramp, which results in a different time needed for the signal to cross a comparator’s threshold. Since a digital counter starts counting at the beginning of the integration process, at the triggering of the comparator, the counter content will be a digital representation of the input signal. Another commonly used ADC type is the Successive Approximation Register (SAR) converter [7,18,34,51], in which the subsequent decisions of a comparator about the polarity of the AFEs output with respect to a reference are processed to obtain IR-dependent digital codes. This approach features, particularly fast conversions, but its resolution is typically limited by the presence of thermal noise (10). To overcome this issue, an oversampling approach can be considered by employing ∑Δ converters operating in incremental mode [52] in accordance with the multiplexing requirements discussed before.

## 5. Conclusions

This paper reviewed uncooled micro-bolometer IRFPAs, at first showing and discussing their structure and operation, then providing some useful metrics to effectively evaluate their performance and lastly surveying the ROIC architecture possibilities. Considering that a performance comparison with the literature is not straightforward because very few common metrics are reported in state-of-the-art works, the goal of this review was to categorize and illustrate the most commonly used ROICs, highlighting the common traits of the IRFPAs developed in the last two decades.

In conclusion, micro-bolometers can be considered reliable and efficient devices to be employed for thermal imaging purposes. Their high sensitivity, fast response time and low power consumption make them ideal for a great variety of applications, as discussed in Section 1. While there are still some limitations to be addressed, such as a relatively low frame rate, the continual advancements in this field show great promise for future improvements especially taking mobile applications into account since the operation of these devices is uncooled; this will drive the research in this field toward improved systems in terms of power consumption, of pixel pitch and, consequently, of cost-effectiveness. 

## Data Availability

No new data were created or analyzed in this study. Data sharing is not applicable to this review.

## Abbreviations

The following abbreviations are used in this manuscript:

AFE	Analog Front End
a-Si	amorphous-Silicon
ADC	Analog-to-Digital Converter
BEQ	Bias EQualization
CCD	Charge-Coupled Device
CDS	Correlated Double Sampling
CNT	Carbon NanoTube
CMOS	Complementary Metal-Oxide-Semiconductor
CTIA	Capacitive TransImpedence Amplifier
DAC	Digital-to-Analog-Converter
EMI	ElectroMagnetic Interference
FPN	Fixed Pattern Noise
IC	Integrated Circuit
IR	InfraRed
IRFPA	InfraRed Focal Plane Array
MD-CDS	Multiple Digital Correlated Double Sampling
MEMS	Micro-Electro-Mechanical System
NEP	Noise at Equivalent Power
NETD	Noise at Equivalent Temperature Difference
PSD	Power Spectral Density
PVT	Process Voltage Temperature
ROIC	ReadOut Integrated Circuit
SAR	Successive Approximation Register
TCR	Temperature Coefficient of Resistance
TiO_x_	Titanium Oxide
VO_x_	Vanadium Oxide
ZTC	Zero-Temperature-Coefficient
